# JUMP: replicability analysis of high-throughput experiments with applications to spatial transcriptomic studies

**DOI:** 10.1093/bioinformatics/btad366

**Published:** 2023-06-05

**Authors:** Pengfei Lyu, Yan Li, Xiaoquan Wen, Hongyuan Cao

**Affiliations:** Department of Statistics, Florida State University, 600 W College AVE, Tallahassee, FL 32306, United States; School of Mathematics, Jilin University, 2699 Qianjin ST, Changchun, Jilin 130012, China; Department of Biostatistics, University of Michigan, Ann Arbor, MI 48109, United States; Department of Statistics, Florida State University, 600 W College AVE, Tallahassee, FL 32306, United States; School of Mathematics, Jilin University, 2699 Qianjin ST, Changchun, Jilin 130012, China

## Abstract

**Motivation:**

Replicability is the cornerstone of scientific research. The current statistical method for high-dimensional replicability analysis either cannot control the false discovery rate (FDR) or is too conservative.

**Results:**

We propose a statistical method, JUMP, for the high-dimensional replicability analysis of two studies. The input is a high-dimensional paired sequence of *p*-values from two studies and the test statistic is the maximum of *p*-values of the pair. JUMP uses four states of the *p*-value pairs to indicate whether they are null or non-null. Conditional on the hidden states, JUMP computes the cumulative distribution function of the maximum of *p*-values for each state to conservatively approximate the probability of rejection under the composite null of replicability. JUMP estimates unknown parameters and uses a step-up procedure to control FDR. By incorporating different states of composite null, JUMP achieves a substantial power gain over existing methods while controlling the FDR. Analyzing two pairs of spatially resolved transcriptomic datasets, JUMP makes biological discoveries that otherwise cannot be obtained by using existing methods.

**Availability and implementation:**

An R package JUMP implementing the JUMP method is available on CRAN (https://CRAN.R-project.org/package=JUMP).

## 1 Introduction

Replicability is the cornerstone of modern scientific research. We study conceptual replicability, where consistent results are obtained from studies using different procedures and populations that target the same scientific questions. Replicability is related to but different from meta-analysis. Both approaches look at cross-experiment summaries. In a meta-analysis, the null hypothesis is that there is no effect in all studies. On the other hand, in replicability analysis, the alternative hypothesis is that the effects exist in all studies and the null hypothesis is a composite null, i.e. at least one study does not have an effect. Methods designed for meta-analysis, such as Fisher’s method (Fisher 1925), the Šidák’s method (Šidák 1967), the Lancaster’s method (Lancaster 1961), and the minimum of *p*-values are not applicable for replicability analysis.

We focus on the replicability analysis of genomic data produced by high-throughput experiments (Li *et al.* 2011, Philtron *et al.* 2018, Hung and Fithian 2020, Bogomolov and Heller 2022). In a high-throughput experiment, many candidates are evaluated for their association with a biological feature of interest, and those with significant associations are identified for further analysis. We aim to simultaneously identify replicable features from high-throughput experiments in multiple studies. To analyze a single high-throughput experiment, an acute problem is the multiple comparison. A classic method for multiple comparisons is the false discovery rate (FDR) control approach proposed in Benjamini and Hochberg (1995) (BH). The FDR is defined as the expected value of the ratio of false rejections over total rejections. Suppose we have *m* hypotheses. The BH procedure works as follows. First, order *p*-values $p_{\left(1\right)}\leq \ldots \leq p_{\left(m\right)}.$ Second, find the largest $i_{0}$ such that $p_{\left(i_{0}\right)}\leq \alpha i_{0}/m.$ Third, reject hypotheses corresponding to $p_{\left(1\right)},\ldots ,p_{\left(i_{0}\right)}.$ Under the assumption that *p*-values from the null are independent and follow standard uniform distribution, FDR is controlled at level $\alpha \pi_{0},$ where $\pi_{0}$ is the proportion of null hypotheses. The BH procedure is robust under positive dependence among *p*-values under the null (Benjamini and Yekutieli 2001).

When we have two studies, an *ad hoc* approach for replicability analysis is to first compute *p*-values for each study. Multiple comparison methods such as BH can be used to claim significance in each study. Replicable findings are those that are significant in both studies. This approach cannot control FDR (Bogomolov and Heller 2013). Intuitively speaking, if there is no danger that a multiple testing procedure produces false positives, then this *ad hoc* approach would work. However, multiple testing procedures have a non-zero probability of making false positives unless the procedure does not reject. Thus, an approach that provides control over false positives in each study separately does not guarantee control of overall false positives to test replicability (Bogomolov and Heller 2013). The partial conjunction approach in Benjamini *et al.* (2009) suggests applying a multiple testing procedure, such as BH, on the maximum of *p*-values across two studies. This provides effective FDR control yet the power is low.

In this article, we propose a joint super-uniform maximum *p*-value (JUMP) method for high-dimensional replicability analysis. The null hypothesis consists of three states. The original maximum of *p*-values does not incorporate different states of the null and has a cumulative distribution function far smaller than that of a standard uniform distribution, causing power loss. We use four states for the *p*-value pairs indicating whether they are null or non-null (Chung *et al.* 2014). Conditional on the hidden states, we compute the cumulative distribution function of the maximum of *p*-values for each state separately. Combining with the proportion estimation for each state, we get a conservative approximation of the cumulative distribution function of the maximum of *p*-values under replicability null. To estimate the proportion of each state, we extend the proportion of null estimation in Storey *et al.* (2004) from the two-group model to a four-group model. We require *p*-values in each sequence to follow a standard uniform distribution under the null. This assumption is needed for the BH procedure and Storey *et al.* (2004)’s method of the proportion of null estimation. A step-up procedure is developed to control FDR. By incorporating the composite null feature of replicability analysis, we achieve a substantial power gain. The computation is scalable with a computational cost similar to that of BH.

As proof of concept, we apply JUMP to the replicability analysis of spatial transcriptomic studies. Spatially resolved transcriptomics (SRT) links the transcriptomes to their cellular locations, providing a comprehensive understanding of gene expression profiles in the spatial context of biological systems (Kleino *et al.* 2022). An important first step toward characterizing the spatial transcriptomic landscape of complex tissues is identifying replicable spatially variable genes (SVGs), genes that have clustered signals in the two-dimensional space for spatial transcriptomic data. For each study, we can apply existing SVG detection methods to get *p*-values (Edsgärd *et al.* 2018, Svensson *et al.* 2018, Sun *et al.* 2020, Hu *et al.* 2021, Zhu *et al.* 2021). The input to our method is a paired *p*-value sequence collected from different tissue sections: mouse olfactory bulb data measured with ST technology (Ståhl *et al.* 2016) and 10× Visium technology; mouse cerebellum data measured with Slide-seq technology (Rodriques *et al.* 2019), and Slide-seqV2 technology (Stickels *et al.* 2021). We show that at the same FDR level, JUMP identifies important replicable SVGs that would otherwise be missed by using existing methods.

## 2 Materials and methods

### 2.1 Background and notations

Suppose we have *m* genes common to two studies. We are interested in identifying genes that display replicable expression patterns. The input of the replicability analysis is a pair of *p*-values from two studies $(p_{1i},p_{2i}),i=1,\ldots ,m.$ The null hypothesis for *i*th gene states that it does not show any replicable expression pattern. Let $\theta_{ji}$ denote underlying state of *i*th gene in study *j* (j=1,2), where $\theta_{ji}=1$ indicates *i*th gene is significant in study *j* and $\theta_{ji}=0$ otherwise. A four-group model is assumed for the paired *p*-value sequence, i.e. where $f_{0}$ is the density function of *p*-values under the null, and $f_{1}$ and $f_{2}$ denote the non-null density functions of *p*-values from study 1 and study 2, respectively. Let $\tau_{i}=(\theta_{1i},\theta_{2i}),i=1,\ldots ,m$ denote the joint hidden states across two studies. Then $\tau_{i}\in \{(0,0),(0,1),(1,0),(1,1)\}$ with $P(\tau_{i}=(k,l))=\xi_{kl}$ for k,l∈{0,1}, and $\sum_{k,l}\xi_{kl}=1$. Here $\xi_{00}$, $\xi_{01}$, $\xi_{10}$, and $\xi_{11}$ denote the probabilities of hidden states (0,0),(0,1),(1,0) and (1,1), respectively. Based on this four-group model, the replicability null hypothesis for *i*th gene can be defined as where the *i*th gene is replicable if it shows expression patterns in both study 1 and study 2. For simplicity, we denote the hidden state of $H_{i0}$ as $h_{i}$, where $h_{i}=0$ indicates $H_{i0}$ is true and $h_{i}=1$ otherwise. Hence $P(h_{i}=0)=\xi_{00}+\xi_{01}+\xi_{10}$ and $P(h_{i}=1)=\xi_{11}$.


$$\begin{array}{c} p_{1i}|\theta_{1i}∼(1-\theta_{1i})f_{0}+\theta_{1i}f_{1},\\ p_{2i}|\theta_{2i}∼(1-\theta_{2i})f_{0}+\theta_{2i}f_{2},\;i=1,\ldots ,m, \end{array}$$

$$H_{i0}:\tau_{i}\in \{(0,0),(0,1),(1,0)\},i=1,\ldots ,m,$$

### 2.2 JUMP for replicability analysis

**Figure 1:**
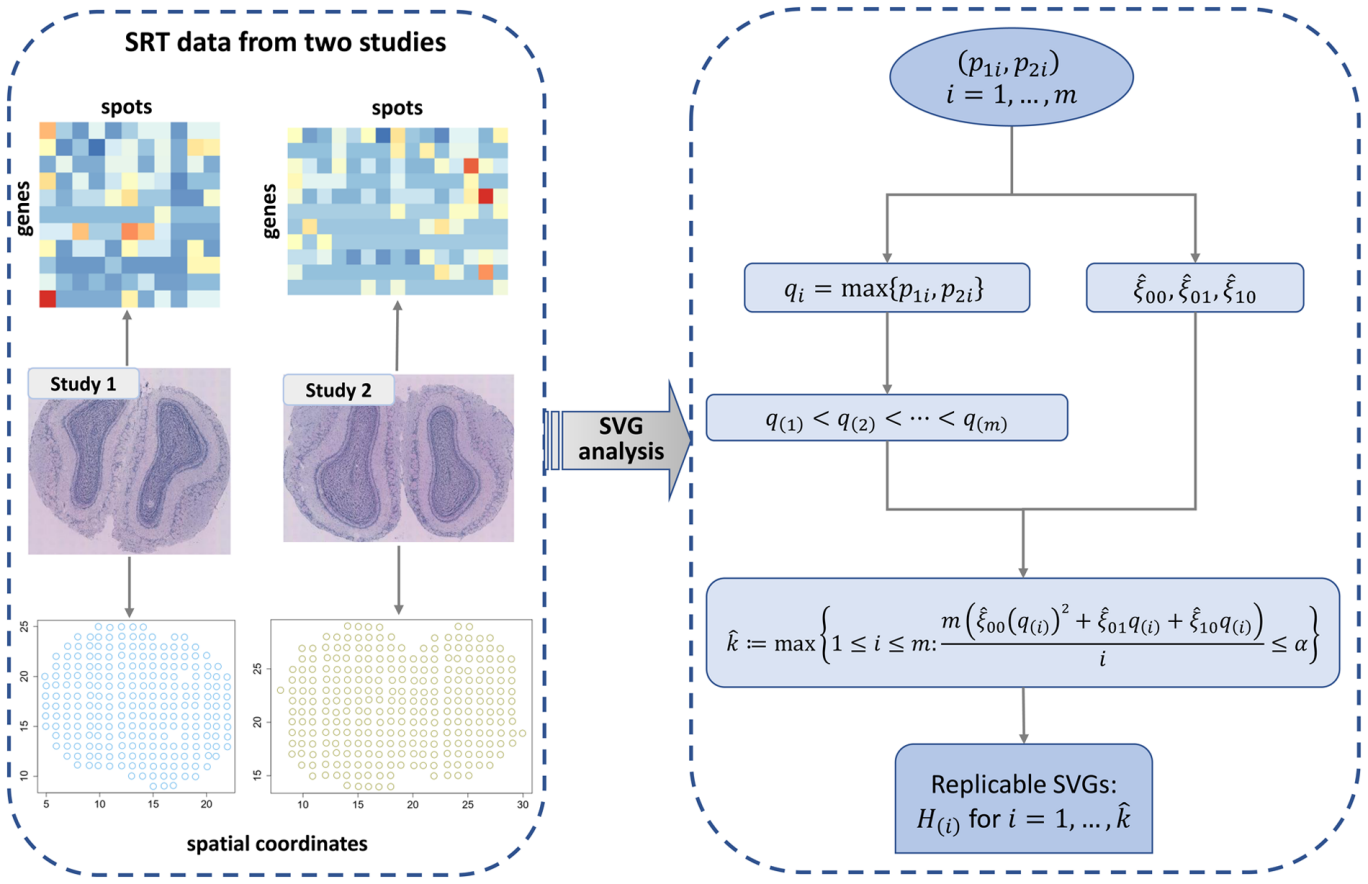
Schematic of JUMP for identifying replicable SVGs from two SRT studies.

The schematic of JUMP for identifying replicable SVGs from two SRT studies is shown in Fig. 1. After obtaining paired *p*-values from two studies, we define the maximum *p*-values as


$$q_{i}=\max\{p_{1i},p_{2i}\},\;i=1,\ldots ,m.$$

Under the assumption that $f_{0}$ follows the standard uniform distribution, we have where we use the assumption that conditional on the joint hidden states, two *p*-value sequences are independent.


$$\begin{array}{c} P(q_{i}\leq t|\tau_{i}=(0,0))=t^{2},\\ P(q_{i}\leq t|\tau_{i}=(0,1))\leq t,\\ P(q_{i}\leq t|\tau_{i}=(1,0))\leq t,\;i=1,\ldots ,m, \end{array}$$

Under the replicable null, $q_{i}$ follows a two-group mixture model where $g_{0}$ and $g_{1}$ denote the density function of $q_{i}$ under replicable null and non-null, respectively. For any t∈(0,1), we compute the cumulative distribution function of $q_{i}$ under replicable null as follows.


$$q_{i}|h_{i}∼(1-h_{i})g_{0}+h_{i}g_{1},$$

$$\begin{array}{c} P(q_{i}\leq t|h_{i}=0)\\ =\frac{P(q_{i}\leq t,h_{i}=0)}{P(h_{i}=0)}\\ =\frac{\xi_{00}P\left(q_{i}\leq t|\tau_{i}=(0,0)\right)}{\xi_{00}+\xi_{01}+\xi_{10}}+\frac{\xi_{01}P\left(q_{i}\leq t|\tau_{i}=(0,1)\right)}{\xi_{00}+\xi_{01}+\xi_{10}}\\ +\frac{\xi_{10}P\left(q_{i}\leq t|\tau_{i}=(1,0)\right)}{\xi_{00}+\xi_{01}+\xi_{10}}\\ \leq \frac{\xi_{00}t^{2}+(\xi_{01}+\xi_{10})t}{\xi_{00}+\xi_{01}+\xi_{10}} \end{array}$$

Denote


(1) $$G(t)=\frac{\xi_{00}t^{2}+(\xi_{01}+\xi_{10})t}{\xi_{00}+\xi_{01}+\xi_{10}},$$

we have


$$G(t)-t=\frac{\xi_{00}}{\xi_{00}+\xi_{01}+\xi_{10}}t(t-1)\leq 0.$$

Hence $P(q_{i}\leq t|h_{i}=0)\leq t$, which means that $q_{i}$ follows a super-uniform distribution under the replicability null. This verifies that the vanilla maximum *p*-value method has valid FDR control.

For t∈(0,1), the number of rejections is $R(t)=\sum_{i=1}^{m}I\{q_{i}\leq t\}$, and the number of false rejections is bounded by $m(\xi_{00}+\xi_{01}+\xi_{10})G(t).$ At threshold *t*, we have a conservative estimate of FDR by


$${\text{FDR}}^{*}(t)=\frac{m(\xi_{00}+\xi_{01}+\xi_{10})G(t)}{R(t)\vee 1}.$$

In the oracle case that we know $\xi_{00},\xi_{01}$ and $\xi_{10}$, at FDR level α, let


(2) $$t_{m}=\sup\{t\in (0,1):{\text{FDR}}^{*}(t)\leq \alpha \}.$$

Reject $H_{i}$ if $q_{i}\leq t_{m}$.

### 2.3 Estimation of unknowns

As $\xi_{00},\xi_{01},$ and $\xi_{10}$ are unknown in practice, we provide their estimates in this section. Following Storey (2002) and Storey *et al.* (2004), in study *j*, the proportion of null hypotheses, $\pi_{0}^{\left(j\right)}$, can be estimated by


$${\hat{\pi }}_{0}^{\left(j\right)}(\lambda_{j})=\frac{\sum_{i=1}^{m}I\{p_{ji}\geq \lambda_{j}\}}{m(1-\lambda_{j})},j=1,2.$$

Similarly, we estimate $\xi_{00}$ as where $\lambda_{1},\lambda_{2}$, and $\lambda_{3}$ are tuning parameters. We use the smoothing method provided in Storey and Tibshirani (2003) to select tuning parameters. Please see Supplementary Note A for more details. We have


$${\hat{\xi }}_{00}(\lambda_{3})=\frac{\sum_{i=1}^{m}I\{p_{1i}\geq \lambda_{3},p_{2i}\geq \lambda_{3}\}}{m{\left(1-\lambda_{3}\right)}^{2}},$$

$${\hat{\xi }}_{01}={\hat{\pi }}_{0}^{\left(1\right)}-{\hat{\xi }}_{00},\;{\hat{\xi }}_{10}={\hat{\pi }}_{0}^{\left(2\right)}-{\hat{\xi }}_{00}.$$

We estimate ${\text{FDR}}^{*}(t)$ by


$${\hat{\text{FDR}}}^{*}(t)=\frac{m({\hat{\xi }}_{00}t^{2}+{\hat{\xi }}_{01}t+{\hat{\xi }}_{10}t)}{R(t)\vee 1}.$$

The data-adaptive thresholding is


(3) $${\hat{t}}_{m}=\sup\{t\in (0,1):{\hat{\text{FDR}}}^{*}(t)\leq \alpha \}.$$

We claim the replicability of *i*th gene if $q_{i}\leq {\hat{t}}_{m}.$

This is equivalent to the following step-up procedure. First, order the maximum *p*-values $q_{\left(1\right)}\leq \ldots \leq q_{\left(m\right)}$. Second, find


(4) $$\hat{k}:=\max\left\{1\leq i\leq m:{\hat{\text{FDR}}}^{*}\left(q_{\left(i\right)}\right)\leq \alpha \right\}.$$

Reject $H_{\left(i\right)}$ for $i=1,\ldots ,\hat{k},$ where $H_{\left(i\right)}$ corresponds to $q_{\left(i\right)}.$

The key to the power gain is to incorporate different states in the composite null. This is similar in spirit to plugging in the proportion of the null hypothesis with a single *p*-value sequence in Storey (2002).

## 3 Results

In this section, we evaluate the FDR control and power of JUMP via simulations and conduct data analysis to identify replicable SVGs from two pairs of SRT datasets measured with different technologies.

### 3.1 Simulation studies

We conducted extensive simulation studies to evaluate the performance of different methods. Power is defined as the expectation of true replicable discoveries over the total number of non-null hypotheses. We compare JUMP with *ad hoc* BH, MaxP, radjust (Bogomolov and Heller 2018), MaRR (Philtron *et al.* 2018), and IDR (Li *et al.* 2011) for replicability analysis. In addition, we used two meta-analysis methods, Šidák’s method (Šidák 1967) and Lancaster’s method (Lancaster 1961), that combine *p*-values across two studies. We applied the BH procedure (Benjamini and Hochberg 1995) on the aggregated *p*-values to show they are not suitable for replicability analysis. Detailed description of different methods can be found in Supplementary Note B.

**Figure 2:**
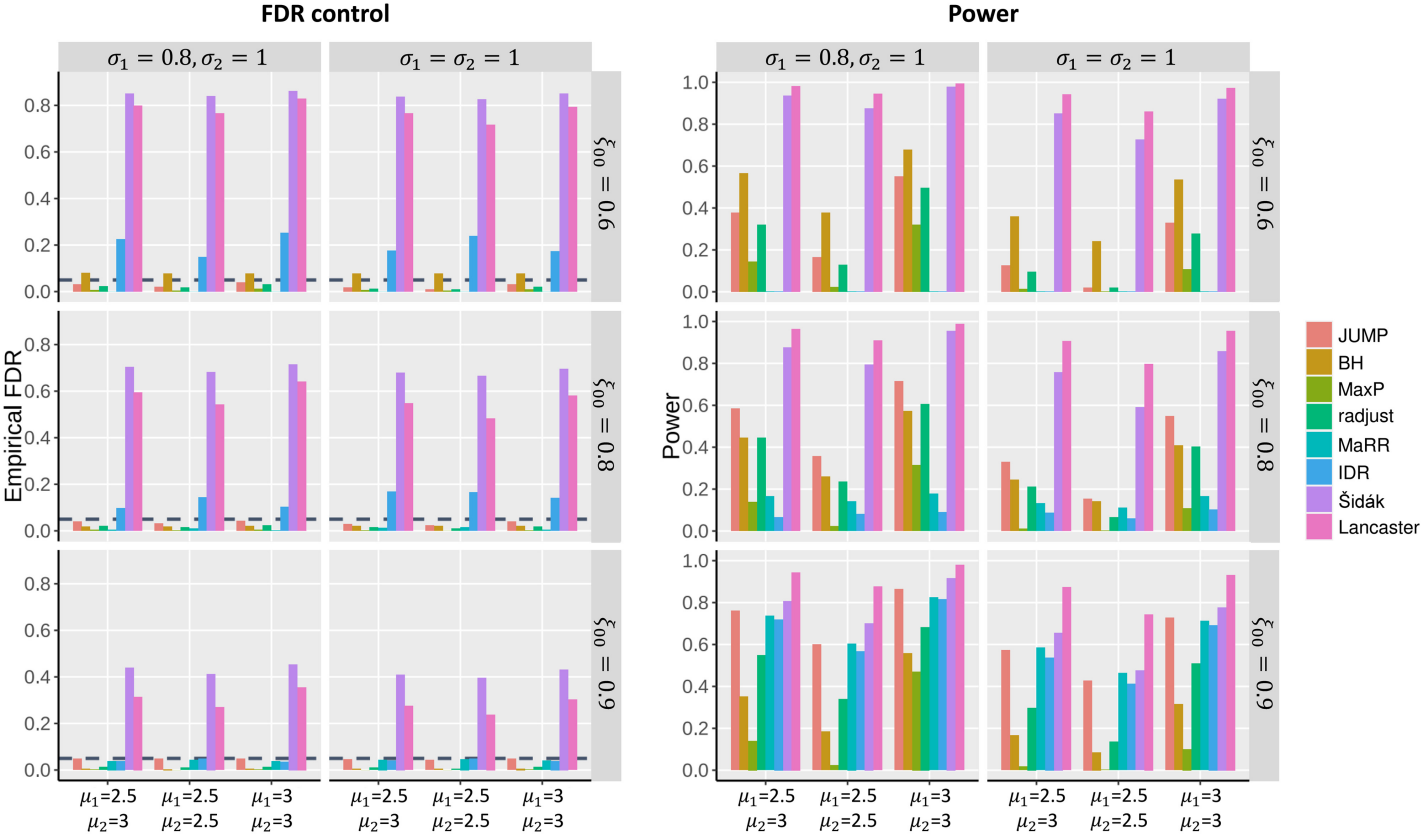
FDR control and power comparison of different methods in simulation studies. $m=10\;000,\;\xi_{11}=0.05$ and $\xi_{01}=\xi_{10}$. Each row corresponds to different $\xi_{00}$. Each column corresponds to different standard deviations. In each panel, the empirical FDR for different methods was calculated over 100 replications at a target FDR level of 0.05 (horizontal dashed line in the plots) for different non-null settings (left: $\mu_{1}=2.5,\mu_{2}=3$; middle: $\mu_{1}=\mu_{2}=2.5$; right: $\mu_{1}=\mu_{2}=3$).

In each simulation, states of genes in two studies, $\theta_{1i}$ and $\theta_{2i}$, were generated from a multinomial distribution with probabilities, $P(\theta_{1i}=k,\theta_{2i}=l)=\xi_{kl},k,l\in \{0,1\}$, for pre-specified $\xi_{00},\xi_{01},\xi_{10}$, and $\xi_{11}$. Denote $N(\mu ,\sigma^{2})$ as a normal distribution with mean μ and standard deviation σ. In simulation study 1, we independently generated summary statistic $X_{ji}∼N(\mu_{j},\sigma_{j}^{2})$ for *i*th gene in study *j* (j=1,2), where $\mu_{j}=0$ if $\theta_{ji}=0$, and $\mu_{j}>0$ if $\theta_{ji}=1$. One-sided *p*-values for study *j* were obtained by $p_{ji}=1-\Phi (Z_{ji}),i=1,\ldots ,m$, where $Z_{ji}=X_{ji}/\sigma_{j}$ denotes the *Z*-statistic for the *i*th gene in study *j* and Φ(⋅) denotes the cumulative distribution function of N(0,1). Figure 2 presents the FDR control and power comparison of different methods under the setting of $m=10,000,\xi_{11}=0.05$ and $\xi_{01}=\xi_{10}$ over different values of $\xi_{00},\mu_{j}$ and $\sigma_{j}$, j=1,2. For a given value of $\xi_{00}$, corresponding $\xi_{01}$ and $\xi_{10}$ can be obtained by $\xi_{01}=\xi_{10}=(1-\xi_{00}-\xi_{11})/2$. At a target FDR level of 0.05, we observe that the Šidák, the Lancaster, the IDR, and the *ad hoc* BH do not have valid FDR control. The MaxP, radjust, MaRR, and JUMP controlled the FDR at 0.05 across all settings. MaxP is overly conservative, radjust and MaRR have decent power, and JUMP has the highest power across all settings.

We also performed realistic simulations based on Replicate 9 and Replicate 12 of ST datasets measured in mouse olfactory bulb (Ståhl *et al.* 2016). Details of the data generation process and simulation results can be found in Supplementary Note C, Figs S1–S3.

### 3.2 Analysis of mouse olfactory bulb data

**Figure 3:**
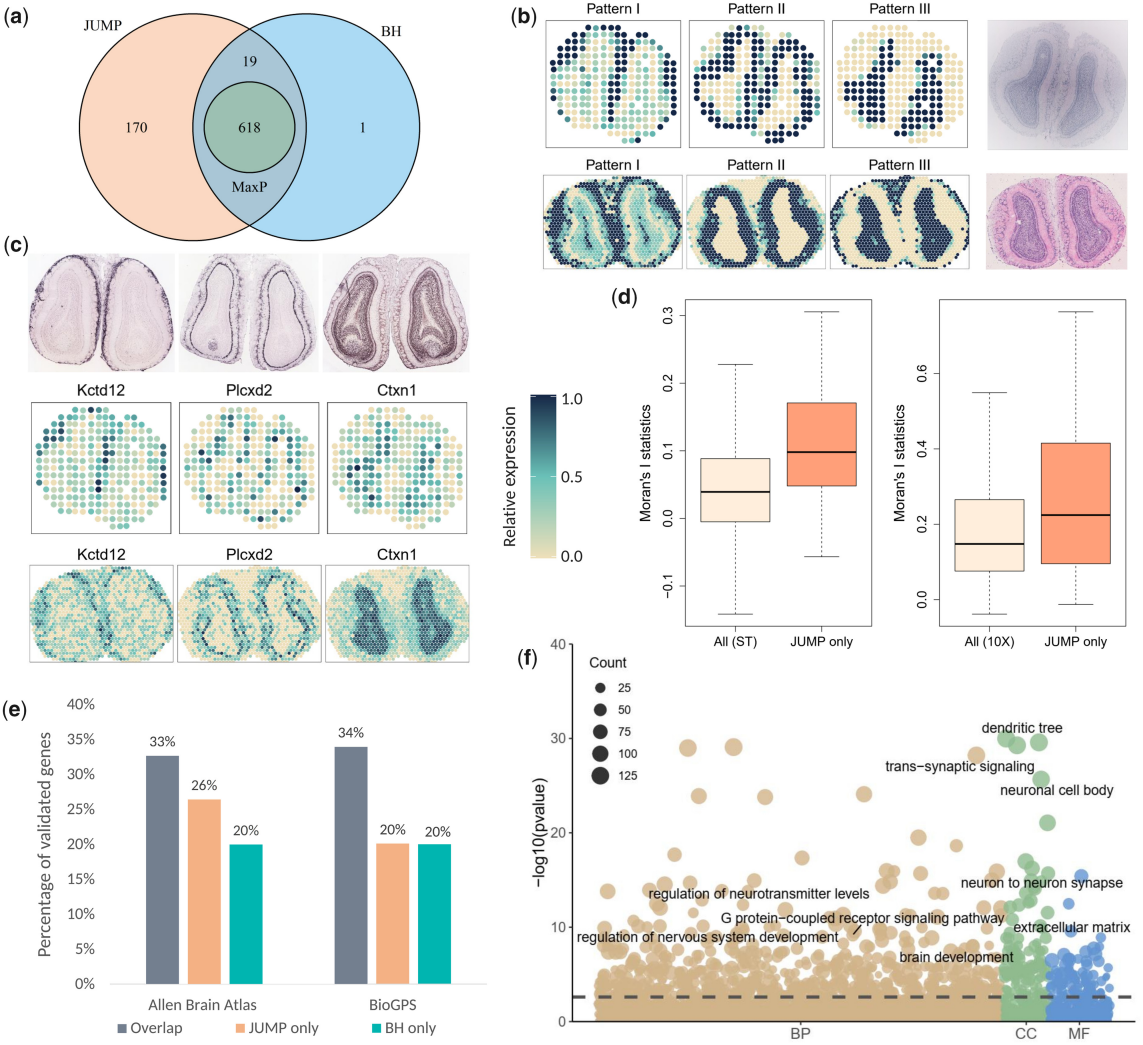
Analysis and validation results of the mouse olfactory bulb data measured with ST technology and 10× Visium technology. (a) Venn diagram shows the number of replicable SVGs identified by different methods at FDR level 0.05 and the intersection of discoveries. (b) Three distinct spatial expression patterns based on the 807 replicable SVGs identified by JUMP in the ST study (top) and 10× Visium study (bottom). Each pattern summarizes the relative expression levels across spatial spots. The corresponding hematoxylin and eosin staining images for the two studies are shown in the right panel. (c) Spatial expression patterns of three representative genes identified by JUMP, corresponding to Patterns I–III, respectively. *In situ* hybridization images of corresponding genes obtained from the Allen Brain Atlas (atlas.brain-map.org) are shown in the top panel. (d) The box plot shows Moran’s *I* statistic of the 189 replicable SVGs additionally identified by JUMP and that of all genes based on the ST study (left) and the 10× Visium study (right). (e) The bar chart displays the number of replicable SVGs additionally identified by JUMP and BH compared to that identified by all three methods. They were validated in two reference gene lists from the Harmonizome database: one from the Allen Brain Atlas dataset and the other from the BioGPS dataset. (f) The bubble plot shows the GO enrichment analysis result of JUMP, including different GO term categories: BP, CC and MF. The horizontal dashed line represents the FDR level 0.01. The size of a bubble represents the counts of corresponding gene sets.

We first analyzed the SRT data from mouse olfactory bulb measured with ST technology (Ståhl *et al.* 2016) and 10× Visium technology. Ståhl *et al.* (2016) published 12 replicates of the mouse olfactory bulb ST datasets on the Spatial Research website (https://www.spatialresearch.org/). We used Replicate 9 for our analysis, which includes 15 284 genes on 237 spots. The 10× Visium dataset was downloaded from the 10× Visium spatial gene expression repository (https://www.10xgenomics.com/resources/datasets) and contains 32 285 genes on 1185 spots. We filtered out genes that are expressed in less than 10% spatial spots and selected spots with at least ten total read counts, resulting in 9547 genes on 236 spots for the ST dataset and 10 680 genes on 1185 spots for the 10× Visium dataset, respectively. We separately analyzed the two datasets using SPARK (Sun *et al.* 2020) to produce *p*-values. Paired *p*-values of 8547 genes common to both studies is the input to the replicability analysis. As can be seen in Fig. 3a, JUMP has higher power than the other two methods. At the FDR level of 0.05, MaxP identified 618 replicable SVGs, which were all detected by BH and JUMP. JUMP identified 807 replicable SVGs, including 637 of the 638 SVGs detected by BH. This is consistent with the simulation results that MaxP is overly conservative and JUMP has higher power.

We clustered the 807 replicable SVGs identified by JUMP into three groups using the hierarchical agglomerative clustering algorithm implemented in the R package *amap* (v0.8-18) and summarized the spatial expression patterns based on the expression level of the genes in corresponding groups. In both studies, the summarized patterns were consistent with three main cell layers in mouse olfactory bulb. In Fig. 3b and Supplementary Fig. S4a, Pattern I corresponds to the glomerular layer, Pattern II corresponds to the mitral layer, and Pattern III corresponds to the granular layer. Spatial expression patterns of three representative SVGs (*Kctd12*, *Plcxd2*, *Ctxn1*) identified by JUMP are presented in Fig. 3c, which correspond to Patterns I–III, respectively and are consistent with the *in situ* hybridization images obtained from the Allen Brain Atlas. For comparison, we also randomly selected 30 genes from the 189 additional findings of JUMP compared to the overlaps of three methods and showed their spatial expression patterns in Supplementary Fig. S5a and b. Moreover, we calculated Moran’s *I* statistic (Moran 1950) of the 189 replicable SVGs additionally identified by JUMP and compared it with that using all 8547 genes to show the autocorrelations of the additional findings by JUMP (Fig. 3d).

To further compare and validate the replicable SVGs identified by different methods, we downloaded two published gene sets from the Harmonizome database (Rouillard *et al.* 2016) consisting of genes related to mouse olfactory bulb as a reference (Fig. 3e). The first gene set was summarized based on three layers (glomerular, mitral, and granular) of the main olfactory bulb from the Allen Brain Atlas adult mouse brain tissue gene expression profiles dataset (Sunkin *et al.* 2013), including 3485 genes with high or low expression in main olfactory bulb relative to other tissues. 33% of the 618 replicable SVGs that were identified by all three methods were validated. Among the 189 replicable SVGs additionally identified by JUMP, 26% were validated in the reference list, whereas only 4 of the 20 SVGs additionally identified by BH were in the same list. The second reference gene set includes 2031 genes differentially expressed in mouse olfactory bulb relative to other cell types and tissues from the BioGPS mouse cell type and tissue gene expression profiles dataset (Wu *et al.* 2013). In addition to the replicable SVGs identified by all three methods (34% validated), 38 of the 189 replicable SVGs only detected by JUMP were in the list, whereas 4 of the 20 replicable SVGs only detected by BH were validated in the same list. These results provide additional biological evidence for the improved power of JUMP.

Additionally, we performed Gene Ontology (GO) enrichment analysis on replicable SVGs identified by JUMP and BH (Fig. 3f). At the FDR level of 0.01, JUMP enriched 846 GO terms and BH enriched 764 GO terms (708 overlaps). Many of the 138 GO terms only identified by JUMP are related to nervous system development and olfactory bulb organization. For instance, transmission of nerve impulse (GO:0019226), neuronal action potential (GO:0019228), and forebrain neuron differentiation (GO:0021879) are closely related to the establishment of axodendritic and dendrodendritic synaptic contacts within the olfactory bulb (Belluzzi *et al.* 2003); GABA-ergic synapse (GO:0098982) plays a key role in the organization of olfactory bulb (Hanson *et al.* 2020); GO terms GO:0045744 and GO:0002029 are related to G protein-coupled receptor, which can be encoded by odorant receptor genes differentially expressed at conserved positions in the olfactory bulb (Katidou *et al.* 2018).

### 3.3 Analysis of mouse cerebellum data

We next analyzed the SRT data from mouse cerebellum measured with Slide-seq technology (Rodriques *et al.* 2019) and Slide-seqV2 technology (Stickels *et al.* 2021). The two datasets were obtained from Broad Institute’s single-cell repository (https://singlecell.broadinstitute.org/single_cell) with IDs SCP354 and SCP948, respectively. The Slide-seq dataset (file ‘Puck_180430_6’) contains 18 671 genes on 25 551 beads. We filtered out beads with total counts less than 50. The Slide-seqV2 dataset contains 23 096 genes on 39 496 beads. We cropped regions of interest by filtering out beads with total counts less than 100. Mitochondrial genes and genes that are not expressed in any locations were filtered out from the two datasets, resulting in 17 481 genes on 14 667 beads for the Slide-seq data and 20 117 genes on 11 626 beads for the Slide-seqV2 data. After applying the SPARK-X method (Zhu *et al.* 2021) on the two datasets separately, we obtained two sequences of *p*-values and matched them by gene. We used the paired *p*-values for 16 519 genes common to both studies as input for the replicability analysis of SVGs. At FDR level 0.05, MaxP identified 279 replicable SVGs, which were all identified by BH and JUMP. JUMP detected all BH findings (394) and identified 54 additional replicable SVGs.

**Figure 4:**
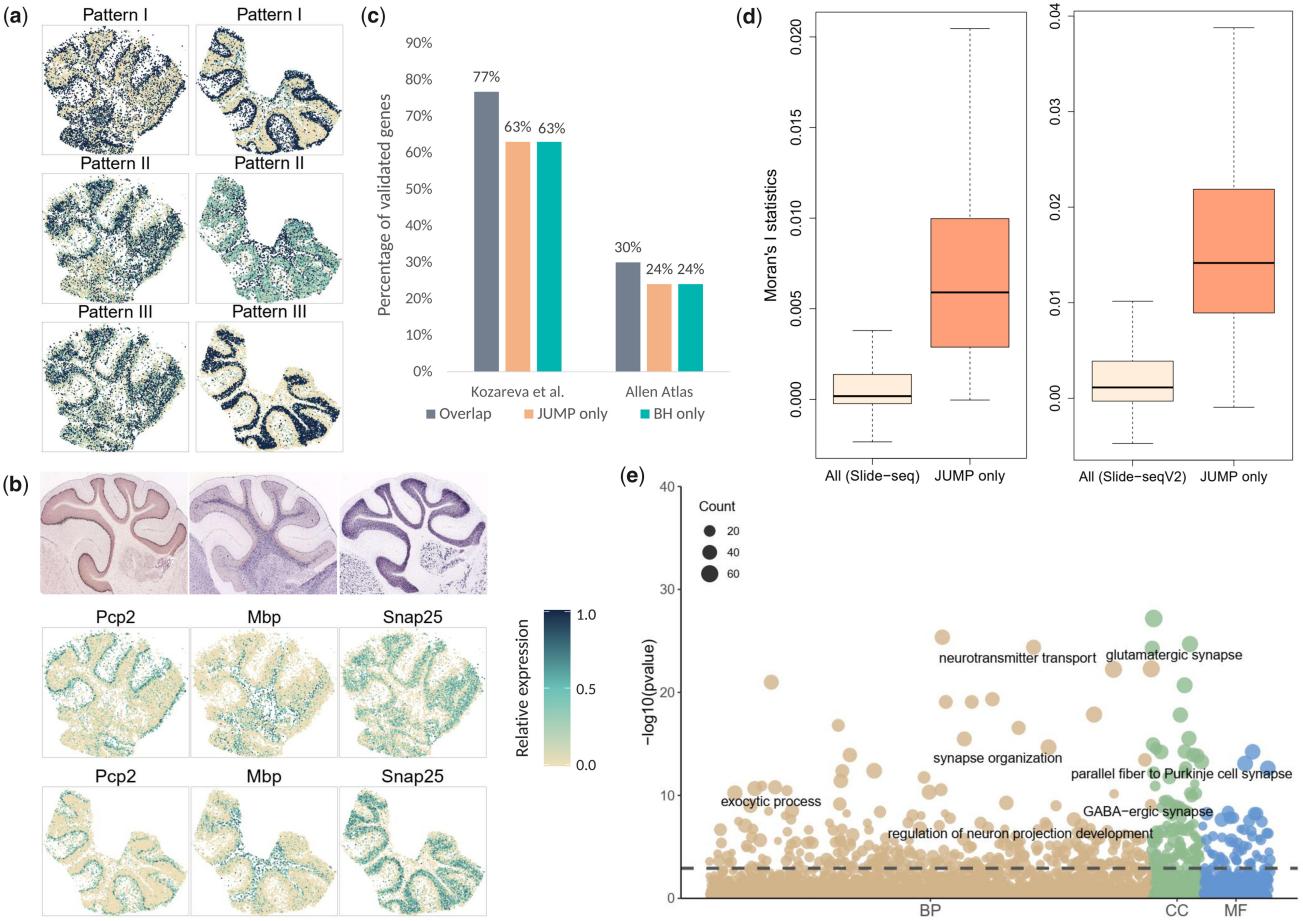
Analysis and validation results of the mouse cerebellum data measured with Slide-seq technology and Slide-seqV2 technology. (a) Three distinct spatial expression patterns based on the 448 replicable SVGs identified by JUMP in the Slide-seq study (left) and Slide-seqV2 study (right). Each pattern summarizes the relative expression levels across spatial spots. (b) Spatial expression patterns of three representative genes identified by JUMP, corresponding to Patterns I–III, respectively. *In situ* hybridization images of corresponding genes obtained from the Allen Brain Atlas (atlas.brain-map.org) are shown in the top panel. (c) The bar chart displays the number of replicable SVGs additionally identified by JUMP and BH compared to that identified by all three methods. They were validated in two reference gene lists: one from Kozareva *et al.* (2021) and the other from the Allen Brain Atlas dataset summarized in the Harmonizome database. (d) The box plot shows Moran’s *I* statistic of the 169 replicable SVGs additionally identified by JUMP and that of all genes based on the Slide-seq study (left) and the Slide-seqV2 study (right). (e) The bubble plot shows the GO enrichment analysis result of JUMP, including different GO term categories: BP, CC and MF. The horizontal dashed line represents the FDR level 0.01. The size of a bubble represents the counts of corresponding gene sets.

We first examined the spatial expression patterns of the 448 replicable SVGs identified by JUMP by clustering them into three groups showing distinct spatial expression patterns (Fig. 4a). In both Slide-seq (left) and Slide-seqV2 (right) datasets, the 448 SVGs showed consistent patterns: Pattern I and Pattern III correspond to the purkinje cell layer and granular cell layer, respectively; and Pattern II corresponds to other cell layers. Three representative genes identified by JUMP (*Pcp2*, *Mbp*, and *Snap25*) exhibited corresponding spatial expression patterns, which were validated by *in situ* hybridization in the Allen Brain Atlas (Fig. 4b). Supplementary Figure S6a and b displays spatial expression patterns of 24 genes randomly selected from the 169 replicable SVGs identified by JUMP in addition to genes identified by all three methods. As shown in Fig. 4d, the additional spatial autocorrelations revealed by JUMP were further confirmed by Moran’s *I* (Moran 1950).

We provided two gene sets obtained from published literature to validate the results of different methods. First, we obtained a list of genes that are highly differentially expressed across all clusters in mouse cerebellar cortex from Kozareva *et al.* (2021). We further filtered out genes with absolute log fold change smaller than 0.05 and obtained 3976 genes for the validation. In addition to genes identified by all three methods (77% validated), 107 of the 169 replicable SVGs additionally identified by JUMP were in the list, whereas 73 of the 115 findings by BH were validated. Second, we downloaded three gene sets related to mouse cerebellum in the Allen Brain Atlas datasets (Sunkin *et al.* 2013) from the Harmonizome database (Rouillard *et al.* 2016) and summarized them to a list of 3000 genes that are differentially expressed in mouse cerebellum, cerebellar cortex, and cerebellar hemisphere. Among the 279 SVGs identified by all three methods, 30% were in this gene list. Thirty-nine of the 169 replicable SVGs additionally identified by JUMP were validated, whereas 28 of the 115 SVGs additionally identified by BH were in the same list.

Finally, we performed GO enrichment analysis on the replicable SVGs identified by different methods to evaluate the biological significance additionally identified by JUMP. At the FDR level of 0.01, JUMP enriched 452 GO terms and BH identified 418 GO terms (394 overlaps). Among the 58 GO terms only enriched by JUMP, many are closely related to the structural constitution and functional development of mouse cerebellum, such as GO terms of cerebellar cortex development (GO:0021695), cerebellum development (GO:0021549), central nervous system neuron development (GO:0021954), dendritic transport (GO:0098935), and photoreceptor ribbon synapse (GO:0098684), among others.

## 4 Discussion

We present a new method, JUMP, for identifying replicable features from two high-throughput experiments. Analysis of different SRT studies identifies important replicable SVGs that might otherwise be missed by existing methods. JUMP is simple to implement and computationally scalable to tens of thousands of genes (Supplementary Tables S1 and S2). Moreover, JUMP does not require two SRT studies to have the same spatial alignment or the same tissue thickness. In addition, JUMP is flexible and can accommodate data from other modalities, such as scRNA-seq, ATAC-seq, and CITE-seq, among others.

One limitation of JUMP is that it only identifies replicable features from two high-throughput experiments. If we want to extend it to more than two studies, say *n* studies, we require a $2^{n}$-group model for the data, and the composite null is composed of $\left(2^{n}-1\right)$ hidden joint states whose proportions need to be estimated. We leave this for future research.

## Supplementary Material

btad366_Supplementary_Data

## Data Availability

The mouse olfactory bulb ST dataset can be obtained with file ‘MOB Replicate 9’ in Spatial Research website at https://www.spatialresearch.org/resources-published-datasets/doi-10-1126science-aaf2403/. The mouse olfactory bulb 10× Visium dataset is available in the 10× Visium spatial gene expression repository at https://www.10xgenomics.com/resources/datasets/adult-mouse-olfactory-bulb-1-standard-1. The mouse cerebellum Slide-seq dataset and Slide-seqV2 dataset are provided in the Single Cell Portal at https://singlecell.broadinstitute.org/single_cell with IDs SCP354 (file ‘Puck_180430_6’) and SCP948, respectively.

## References

[btad366-B1] Belluzzi O , Benedusi M, Ackman J et al Electrophysiological differentiation of new neurons in the olfactory bulb. J Neurosci 2003;23:10411–8. 14614100 10.1523/JNEUROSCI.23-32-10411.2003 PMC6741027

[btad366-B2] Benjamini Y , Hochberg Y. Controlling the false discovery rate: a practical and powerful approach to multiple testing. J R Stat Soc Ser B Methodol 1995;57:289–300.

[btad366-B3] Benjamini Y , Yekutieli D. The control of the false discovery rate in multiple testing under dependency. Ann Stat 2001;29:1165–88.

[btad366-B4] Benjamini Y , Heller R, Yekutieli D. Selective inference in complex research. Philos Trans A Math Phys Eng Sci 2009;367:4255–71. 19805444 10.1098/rsta.2009.0127 PMC3263782

[btad366-B5] Bogomolov M , Heller R. Discovering findings that replicate from a primary study of high dimension to a follow-up study. J Am Stat Assoc 2013;108:1480–92.

[btad366-B6] Bogomolov M , Heller R. Assessing replicability of findings across two studies of multiple features. Biometrika 2018;105:505–16.

[btad366-B7] Bogomolov M , Heller R. Replicability across multiple studies. arXiv, arXiv:2210.00522, 2022, preprint: not peer reviewed.

[btad366-B8] Chung D , Yang C, Li C et al Gpa: a statistical approach to prioritizing GWAS results by integrating pleiotropy and annotation. PLoS Genet 2014;10:e1004787. 25393678 10.1371/journal.pgen.1004787 PMC4230845

[btad366-B9] Edsgärd D , Johnsson P, Sandberg R. Identification of spatial expression trends in single-cell gene expression data. Nat Methods 2018;15:339–42. 29553578 10.1038/nmeth.4634 PMC6314435

[btad366-B10] Fisher R. Statistical Methods for Research Workers. Edinburgh: Oliver & Boyd, 1925.

[btad366-B11] Hanson E , Swanson J, Arenkiel BR. Gabaergic input from the basal forebrain promotes the survival of adult-born neurons in the mouse olfactory bulb. Front Neural Circ 2020;14:17. 10.3389/fncir.2020.00017 PMC7190813 32390805

[btad366-B12] Hu J , Li X, Coleman K et al SpaGCN: integrating gene expression, spatial location and histology to identify spatial domains and spatially variable genes by graph convolutional network. Nat Methods 2021;18:1342–51. 34711970 10.1038/s41592-021-01255-8

[btad366-B13] Hung K , Fithian W. Statistical methods for replicability assessment. Ann Appl Stat 2020;14:1063–87.

[btad366-B14] Katidou M , Grosmaitre X, Lin J et al G-protein coupled receptors Mc4r and Drd1a can serve as surrogate odorant receptors in mouse olfactory sensory neurons. Mol Cell Neurosci 2018;88:138–47. 29407371 10.1016/j.mcn.2018.01.010

[btad366-B15] Kleino I , Frolovaitė P, Suomi T et al Computational solutions for spatial transcriptomics. Comput Struct Biotechnol J 2022;20:4870–84. 36147664 10.1016/j.csbj.2022.08.043 PMC9464853

[btad366-B16] Kozareva V , Martin C, Osorno T et al A transcriptomic atlas of mouse cerebellar cortex comprehensively defines cell types. Nature 2021;598:214–9. 34616064 10.1038/s41586-021-03220-z PMC8494635

[btad366-B17] Lancaster H. The combination of probabilities: an application of orthonormal functions. Aust J Stat 1961;3:20–33.

[btad366-B18] Li Q , Brown JB, Huang H et al Measuring reproducibility of high-throughput experiments. Ann Appl Stat 2011;5:1752–79.

[btad366-B19] Moran PA. Notes on continuous stochastic phenomena. Biometrika 1950;37:17–23. 15420245

[btad366-B20] Philtron D , Lyu Y, Li Q et al Maximum rank reproducibility: a nonparametric approach to assessing reproducibility in replicate experiments. J Am Stat Assoc 2018;113:1028–39. 31249430 10.1080/01621459.2017.1397521 PMC6596287

[btad366-B21] Rodriques SG , Stickels RR, Goeva A et al Slide-seq: a scalable technology for measuring genome-wide expression at high spatial resolution. Science 2019;363:1463–7. 30923225 10.1126/science.aaw1219 PMC6927209

[btad366-B22] Rouillard AD , Gundersen GW, Fernandez NF et al The harmonizome: a collection of processed datasets gathered to serve and mine knowledge about genes and proteins. Database 2016;2016:baw100. 27374120 10.1093/database/baw100 PMC4930834

[btad366-B23] Šidák Z. Rectangular confidence regions for the means of multivariate normal distributions. J Am Stat Assoc 1967;62:626–33.

[btad366-B24] Ståhl PL , Salmén F, Vickovic S et al Visualization and analysis of gene expression in tissue sections by spatial transcriptomics. Science 2016;353:78–82. 27365449 10.1126/science.aaf2403

[btad366-B25] Stickels RR , Murray E, Kumar P et al Highly sensitive spatial transcriptomics at near-cellular resolution with slide-seqv2. Nat Biotechnol 2021;39:313–9. 33288904 10.1038/s41587-020-0739-1 PMC8606189

[btad366-B26] Storey JD. A direct approach to false discovery rates. J R Stat Soc Ser B Stat Methodol 2002;64:479–98.

[btad366-B27] Storey JD , Tibshirani R. Statistical significance for genomewide studies. Proc Natl Acad Sci USA 2003;100:9440–5. 12883005 10.1073/pnas.1530509100 PMC170937

[btad366-B28] Storey JD , Taylor JE, Siegmund D. Strong control, conservative point estimation and simultaneous conservative consistency of false discovery rates: a unified approach. J R Stat Soc Ser B Stat Methodol 2004;66:187–205.

[btad366-B29] Sun S , Zhu J, Zhou X. Statistical analysis of spatial expression patterns for spatially resolved transcriptomic studies. Nat Methods 2020;17:193–200. 31988518 10.1038/s41592-019-0701-7 PMC7233129

[btad366-B30] Sunkin SM , Ng L, Lau C et al Allen brain atlas: an integrated spatio-temporal portal for exploring the central nervous system. Nucleic Acids Res 2013;41:D996–1008. 23193282 10.1093/nar/gks1042 PMC3531093

[btad366-B31] Svensson V , Teichmann SA, Stegle O. SpatialDE: identification of spatially variable genes. Nat Methods 2018;15:343–6. 29553579 10.1038/nmeth.4636 PMC6350895

[btad366-B32] Wu C , MacLeod I, Su AI. BioGPS and MyGene.info: organizing online, gene-centric information. Nucleic Acids Res 2013;41:D561–5. 23175613 10.1093/nar/gks1114 PMC3531157

[btad366-B33] Zhu J , Sun S, Zhou X. SPARK-X: non-parametric modeling enables scalable and robust detection of spatial expression patterns for large spatial transcriptomic studies. Genome Biol 2021;22:1–25. 34154649 10.1186/s13059-021-02404-0 PMC8218388

